# Assessment of Early Response to Lung Cancer Chemotherapy by Semiquantitative Analysis of Dynamic Contrast-Enhanced MRI

**DOI:** 10.1155/2022/2669281

**Published:** 2022-07-23

**Authors:** Jiang Zhu, Jian Yun, Kaixiang Wang, Liangqing Liu, Jiangang Zheng, Li Mei, Jianxing Xu

**Affiliations:** ^1^Department of Radiology, Wujin Hospital Affiliated with Jiangsu University, Changzhou 213002, China; ^2^Department of Radiology, The Wujin Clinical College of Xuzhou Medical University, Changzhou 213002, China

## Abstract

**Objective:**

To evaluate the early chemotherapy response in patients with lung cancer using semiquantitative analysis of dynamic contrast-enhanced (DCE) magnetic resonance imaging (MRI).

**Methods:**

Twenty-two patients with lung cancer treated with chemotherapy were subjected to DCE-MRI at two time points: before starting treatment and after one week of therapy. The image data were collected by DCE-MRI, and the semiquantitative parameters including positive enhancement integral (PEI), signal enhancement ratio (SER), maximum slope of increase (MSI), and time to peak (TTP) were calculated. After chemotherapy, the parameters and relevant variations between the responders and nonresponders were compared with Mann–Whitney *U* tests. Student's *t*-test for paired samples was used to evaluate the temporal changes between pre- and posttreatment images.

**Results:**

The patients were categorized as 13 responders and 9 nonresponders based on the tumor response evaluation. After chemotherapy, the PEI, SER, and MSI were significantly increased in responders compared with the pretreatment values (*P* < 0.05), while no obvious decrease in TTP was observed (*P* > 0.05). However, 9 nonresponders showed no significant changes in PEI, SER, MSI, and TTP values, as compared with those of pretreatment (*P* > 0.05). Moreover, the increase of PEI was more dramatically in responders than in nonresponders (*P* < 0.05), but no significantly differences were observed in SER, MSI, and TTP (*P* > 0.05).

**Conclusion:**

Semiquantitative analysis of DCE-MRI could provide a reliable noninvasive method for assessing early chemotherapy response in lung cancer patients.

## 1. Introduction

Chemotherapy is considered as the main regimen in the treatment of advanced lung cancer. Poor efficacy will result in increased toxicity and morbidity. Patients will have undergone toxic therapy without any benefits. Therefore, imaging procedures which can rapidly and accurately evaluate the treatment response are needed. The treatment response is evaluated according to the response evaluation criteria in solid tumors (RECIST), which mainly assesses the changes of tumor size on computed tomography (CT) [1, 2]. Nonetheless, the size of tumor changes lagging than biological and molecular levels [3, 4]. Therefore, explorations of reliable surrogate markers that can indicate treatment response are warranted.

Parameters that describe the tumor microenvironment by demonstrating perfusion and capillary permeability could be obtained by dynamic contrast-enhanced (DCE)-MRI [5, 6]. Moreover, the parameters reflecting tumor blood volume and blood flow can be used as biomarkers in the assessment of tumor vascularity, which is relevant to the chemotherapy response [7–9]. The analytical methods applied to the DCE-MRI data vary from a simple by-eye observation of the time-dependent variation in signal intensity after contrast delivery to the more complicated methods using theoretical pharmacokinetic models [10]. Most of the recent studies have focused on the relationship between tumor response to therapy and quantitative parameters obtained from pharmacokinetic analysis of DCE-MRI [11–16]. Our previous study also found that these quantitative parameters might be as biomarkers in the early assessment of chemotherapy response in lung cancer patients [17].

Semiquantitative measurements derived from a plot of signal intensity with time have been shown to correlate with antiangiogenic treatment for recurrent glioma [18]. Particularly, recent studies have also analyzed that semiquantitative parameters are highly correlated with quantitative parameters generated from DCE-MRI and semiquantitative parameters sometimes may be superior to the counterparts in assessing the therapy response [10, 13, 19]. Therefore, we hypothesized that semiquantitative analysis of DCE-MRI could also be valuable in the evaluation of chemotherapy response in lung cancer. The purpose of this study was to assess the early chemotherapy response in patients with lung cancer using semiquantitative analysis of DCE-MRI.

## 2. Materials and Methods

### 2.1. Subjects

The patients histologically diagnosed as advanced lung cancer (stage IIIB or IV) at our hospital from 1^st^ Feb 2015 to 31^st^ Aug 2017 were enrolled. The inclusion criteria were as follows: (a) the maximum diameter of the mass was ≥3.0 cm, (b) no history of previous therapy, and (c) no known metallic implants or claustrophobia. The exclusion criteria were as follows: (a) with contraindications to gadolinium contrast agent (renal dysfunction or allergy), (b) have been treated with radiotherapy or chemotherapy, (c) treatment discontinued within three months, and (d) poor visualization of the MRI images. The prospective study was approved by the ethics committee of the Affiliated Wujin Hospital of Jiangsu University, and informed written consent was obtained from each patient. Patients were informed of the potential benefits and contraindications in DCE-MRI.

### 2.2. Clinical Treatment and Tumor Response Assessment

All patients underwent at least 3 cycles of cisplatin-based chemotherapy (21 days per cycle). After three cycles of chemotherapy, the treatment response was evaluated based on the RECIST [2] and classified as follows: patients were categorized as responders when all target lesions completely or partially disappeared (lesions decreased ≥30% from baseline diameters) and patients were categorized as nonresponders when the target lesions were relatively stable (<30% reduction or<20% increase from baseline diameters) or have been progressed (≥20% increase from baseline diameters of the original lesions or occurrence of new tumor) [20].

### 2.3. Image Acquisition

Pretreatment and posttreatment MRI images which were taken one week after the start of chemotherapy were performed using a 1.5 T unit (Avanto, Siemens, Erlangen, Germany) with an eight-channel body-phased array coil. First, we took the respiratory-gated T2-weighted axial images and breath-hold T2-weighted coronal images. DCE-MRI sequences were obtained with breath-free T1-weighted VIBE images, which contained 30 scans of 4 s each. Examination protocols of MRI are shown in Table 1. Baseline images were obtained before the contrast media injection. Gadolinium contrast agent (Magnevist, Bayer Schering, Berlin, Germany) was used for contrast enhancement and was injected intravenously using an MR-compatible power injector (Mallinckrodt Optistar, Liebel-Flarsheim, Cincinnati, OH, USA) with a dosage of 0.1 mmol/kg and a rate of 2.5 mL/sec. Then, it was followed by a chasing bolus of 15 mL normal saline administered at the same rate.

### 2.4. Data Analysis

With the help of free-standing workstation (United Imaging Medical Systems, Shanghai, China), the tumor border was codetermined on the dynamic images by two radiologists who has 16 years and 18 years of diagnose experience in MRI, respectively. The regions of interest (ROIs) were manually defined on axial slice which was selected at the level of the maximum diameter on the postcontrast image. The ROIs were drawn along the contours of each tumor. To reduce the partial volume effect, the ROIs were slightly smaller in size than the actual tumor size. Time intensity curves (TIC) were obtained, and positive enhancement integral (PEI), signal enhancement ratio (SER), maximum slope of increase (MSI), and time to peak (TTP) were calculated as previously described [21, 22].

### 2.5. Statistical Analysis

Data were presented as the mean ± standard deviation. Statistical analysis was performed using SPSS software (version 22.0, Chicago, IL). Two-sided *P* < 0.05 was considered to indicate a statistically significant difference. The Mann–Whitney *U* test was used to evaluate the differences between two groups. Student's *t*-test for paired samples was used to evaluate the temporal changes in the parameters between pre- and posttreatment images.

## 3. Results

**Figure 1:**
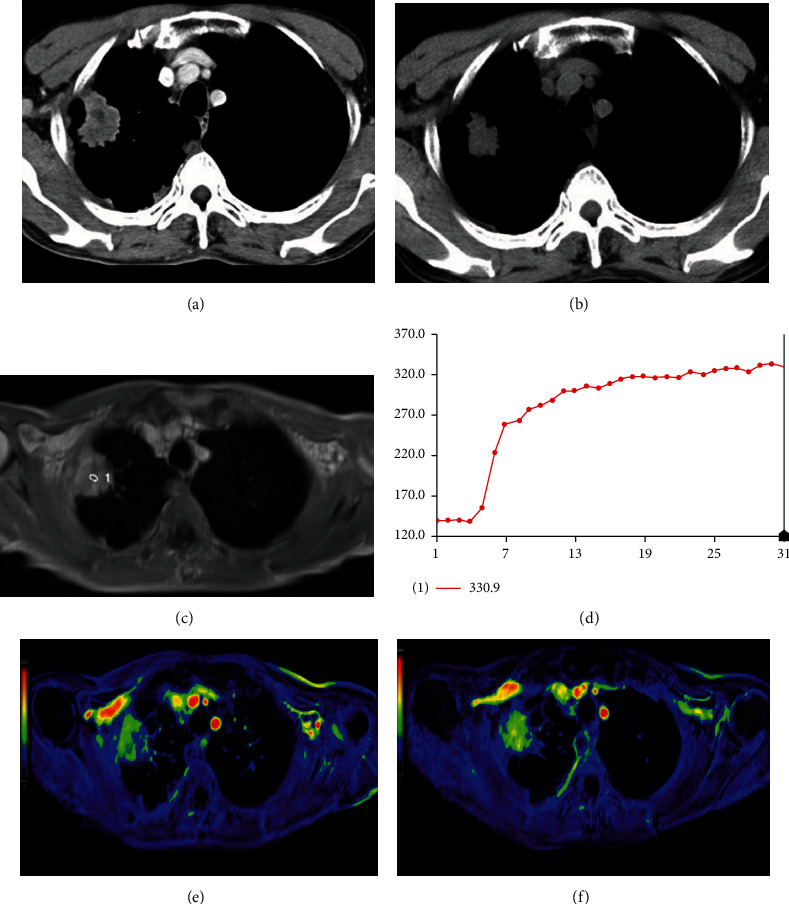
Axial CT scan of a 72-year-old male with lung adenocarcinoma at the right upper lobe before (a) and after (b) three months of chemotherapy. At the maximum transverse dimension, about 31.7% decrease of the tumor size was observed, and the patient was categorized as a responder. The DCE-MRI map (c) and TIC map (d) are shown. Images of color MR PEI mapping before (e) and after (f) one week of the first course of chemotherapy showed that there was an obvious increase in tumor perfusion (the value was 841 vs. 1100).

**Figure 2:**
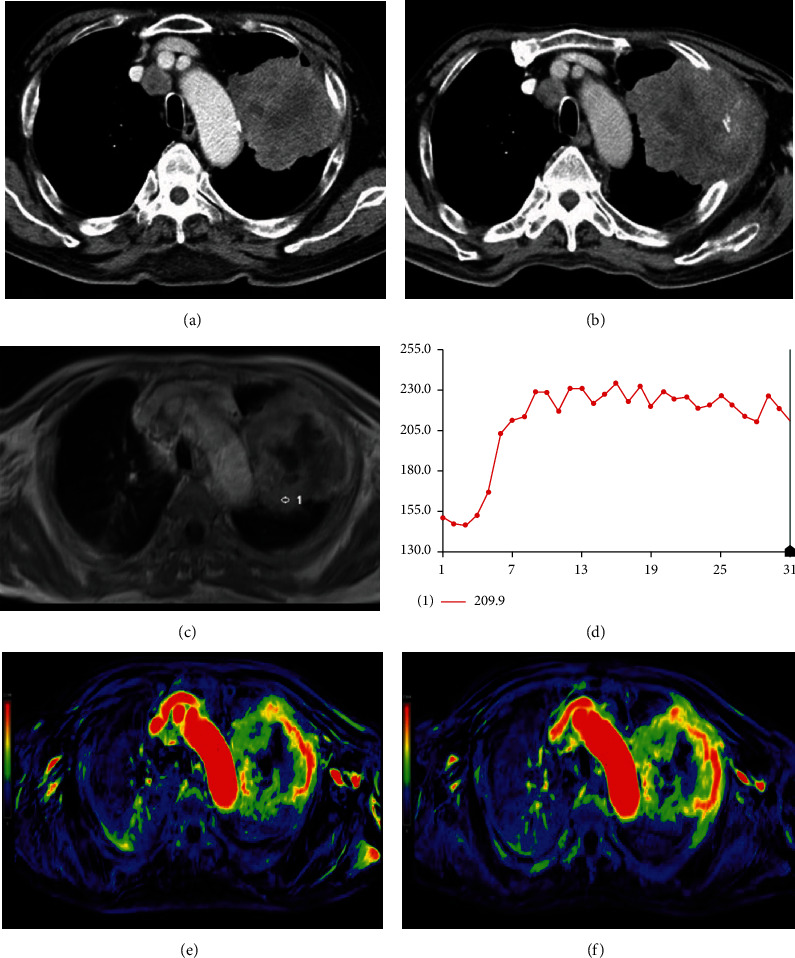
Axial CT scan of a 68-year-old male with lung squamous cell carcinomas at the left upper lobe before (a) and after (b) three months of chemotherapy. At the maximum transverse dimension, about 12.0% increase of the tumor size was observed, and the patient was categorized as a nonresponder. The DCE-MRI map (c) and TIC map (d) are shown. Images of color MR PEI mapping before (e) and after (f) one week of the first course of chemotherapy showed not significant increase in tumor perfusion (the value was 914 vs. 1078).

In this study, we included a total of 25 patients with advanced lung cancer, of which 3 patients were not used for DCE-MRI analysis due to vigorous breathing exercise. Therefore, 22 patients were finally included in this study, including 11 adenocarcinomas, 7 squamous cell carcinomas, and 4 small cell carcinomas. All patients had eligible pretreatment imaging and received chemotherapy, and there were no significant differences in basic clinical characteristics among all patients. Patients' response to chemotherapy was assessed three months after treatment. The results showed that 13 patients were classified as responders and the other 9 patients were classified as nonresponders. After 1 week of chemotherapy, there was no significant difference in the maximum diameter of target lesions between the two groups compared with the value before treatment (*P* = 0.141 and *P* = 0.899). Generate semiquantitative parameters and calculate averages. The detailed parameters of PEI, SER, MSI, and TTP are shown in Table 2 and compared. As shown in Figures 1 and 2, there were no significant differences in baseline PEI, SER, MSI, and TTP values between responders and nonresponders (*P* = 0.500, *P* = 0.063, *P* = 0.474, and *P* = 0.256, respectively).

The 13 responders had significantly higher PEI, SER, and MSI values after chemotherapy (*P* = 0.003, *P* = 0.023, and *P* = 0.046, respectively), while TTP did not decrease significantly (*P* = 0.726). In contrast, PEI, SER, MSI, and TTP values were not significantly changed in the 9 nonresponders compared to pretreatment (*P* = 0.260, *P* = 0.441, *P* = 0.515, and *P* = 0.441, respectively). In addition, the degree of change of these parameters after treatment is shown in Table 3 and compared. The results showed that responders had a more significant increase in PEI than nonresponders (*P* = 0.006), while SER, MSI, and TTP were not statistically significant (*P* = 0.301, *P* = 0.215, and *P* = 0.397, respectively).

## 4. Discussion

In this study, we investigated the chemotherapy response of lung cancer patients with the use of semiquantitative analysis. Our results showed that there was significant increase in PEI, SER, and MSI values in those responders after treatment, as compared with those of pretreatment. And the increased degree of PEI (*Δ*PEI) in responders was significantly different from that in nonresponders. Our preliminary results indicated that the increase of PEI, SER, and MSI values might be associated with early chemotherapy response in patients with lung cancer. Also, *Δ*PEI might be taken as a direct predictive value of chemotherapy efficiency.

Our results were inconsistent with a recent study [9], which reported that these semiquantitative parameters including PEI and MSI were significantly decreased after therapy than those measured before treatment. We speculated that these conflicted results might be due to the limited sample size, tumor heterogeneity, and the acquisition time of posttreatment imaging data. First, the limited number of patients potentially resulted in interpretation bias of the DCE-MRI data. Second, the ROIs were manually defined to avoid partial volume effect. However, it might also fail to reflect tumor heterogeneity, which was rather important in tumor therapy because high heterogeneity is correlated with tumor prognosis [23]. Third, the posttreatment MRI was performed a week after the start of chemotherapy, while it was conducted within three weeks after the whole cycles in previous studies. Although the optimal acquisition timing of DCE-MRI data for therapeutic evaluation has not been defined, previous studies have generally suggested that early MRI readings may be more reliable than the later ones [24], because the change of these parameters were not purely dependent on tumor perfusion but also related to secondary inflammation [25].

Moreover, previous studies have shown that the rich blood flow could be attributed to tumor-associated vasculature and vascularization [26, 27]. Tumors with relatively rich blood flow have higher oxygenation levels, leading to better access to chemotherapy [25]. Many other studies found that increase in enhancement early during radiotherapy was associated with better prognosis [28, 29]. Studies using animal models [30] have also demonstrated the increase of tumor perfusion after radiotherapy. These changes are resulted from radiation-induced tumor microvasculature and the increase of vascular bed per unit tumor volume due to shrinking tumor mass [31–34]. Based on our results, we hypothesized that the short-term increase in these parameters obtained a week after the first course of chemotherapy might be related to higher blood flow, increased oxygenation levels, and better access to chemotherapy. On the other hand, the decrease in these semiquantitative parameters observed after 3 weeks of chemotherapy was associated with tumor hypoxia. The detection of short-term changes by these DCE-MRI parameters proved the ability of this technique to determine treatment-related changes in tumor perfusion. To be sure, clinical studies with a large sample size are desired to confirm these promising results.

In our study, we found no significant changes in TTP after treatment and no significant difference between responders and nonresponders, which is consistent with the previous study. Therefore, though several studies have shown that TTP is promising in distinguishing benign tumors from malignant ones [21, 22], our results indicated that TTP alone could not predict therapy response in lung cancer patients.

In addition, the semiquantitative parameters generated from DCE-MRI data are influenced by several factors, including the dosage and administration rate of the contrast agent, the strength of the magnetic field, reflexivity, respiratory motion artifacts, and the MR sequence being used [19]. Skinner et al. [35] hypothesized that the greatest effects between well-vascularized and necrotic tumor regions were different in contrast agent concentration dynamics. Furthermore, it should be emphasized that these semiquantitative parameters could be influenced by tumor therapy regimens. Different therapy regimens might have played a confounding role in telling responders from nonresponders.

There are several limitations of this study: first, the sample size was small and there was a lack of long-term follow-up. Also, the patients in this study rarely had lymph node metastasis or distant metastasis. We should initiate a larger cohort to validate the benefit of these semiquantitative DCE-MRI parameters in evaluating the therapy response. Second, these parameters were measured on one axial section of the maps, which may not be representative. And the calculated mean values might have overlooked the tumor heterogeneity. Third, the optimal evaluation timing for chemotherapy response remains controversial and consecutive study is needed. Last, it would be better if we conduct a comparison study between semiquantitative and quantitative parameters in this case.

## 5. Conclusion

In conclusion, PEI can be used as a potential biomarker for early assessment of response to chemotherapy in lung cancer. With further validation, these semiquantitative DCE-MRI parameters help to optimize treatment strategies.

## Figures and Tables

**Table 1:** 1.5 T MR imaging sequences and parameters.

	T2WI	T2WI	DCE-MRI
Sequence type	TSE	TSE	VIBE
Field of view (mm)	400 × 400	380 × 300	380 × 300
Acquisition matrix	230 × 256	314 × 320	129 × 96
TR/TE (ms)	385/1.16	2000/85	2.89/1.06
Number of averages	2	3	1
Thickness (mm)	4	7	5
Interslice gap	20%	20%	20%
Slices	30	45	48

TSE: turbo spin echo; VIBE: volumetric interpolated breath-hold examination, 3D-T1WI-GRE (gradient recalled echo); TR: repetition time; TE: echo time.

**Table 2:** Results and comparisons of DCE MR-derived semiquantitative parameters both in responders and nonresponders.

Group	Number	Parameters	Pretreatment	Posttreatment	*Z* value	*P* value
Responders	13	PEI	863.38 ± 301.04	1063.15 ± 331.06	-2.970	0.003∗
		SER	142.85 ± 34.43	164.23 ± 65.58	-1.293	0.023∗
		MSI	380.46 ± 123.17	466.46 ± 142.63	-1.992	0.046∗
		TTP	186.31 ± 56.73	169.62 ± 33.63	-0.350	0.726
Nonresponders	9	PEI	868.78 ± 357.29	747.22 ± 0319.13	-1.125	0.260
		SER	177.67 ± 56.85	192.00 ± 78.13	-0.770	0.441
		MSI	392.11 ± 145.401	400.33 ± 114.35	-0.652	0.515
		TTP	162.11 ± 18.13	158.33 ± 18.16	-0.770	0.441

Values listed are the mean ± standard deviations. ∗*P* < 0.05, 2 related sample Mann–Whitney *U* tests.

**Table 3:** Changes and comparisons of DCE MR-derived semiquantitative parameters between responders and nonresponders.

Parameters	Responders (*n* = 13)	Nonresponders (*n* = 9)	*Z* value	*P* value
PEI	238.23 ± 248.09	−121.56 ± 306.98	-2.504	0.006∗
SER	32.92 ± 43.88	14.33 ± 67.65	-0.167	0.301
MSI	86.00 ± 120.26	8.22 ± 131.25	-0.802	0.215
TTP	−16.69 ± 47.405	−3.78 ± 23.98	-0.267	0.397

Values listed are the mean ± standard deviations. ∗*P* < 0.05, 2 independent sample Mann–Whitney *U* tests.

## Data Availability

The data are available upon request.
